# Comparison of Volatile Profiles of Meads and Related Unifloral Honeys: Traceability Markers

**DOI:** 10.3390/molecules27144558

**Published:** 2022-07-17

**Authors:** Piotr M. Kuś, Sławomir Czabaj, Igor Jerković

**Affiliations:** 1 Department of Pharmacognosy and Herbal Medicines, Faculty of Pharmacy, Wrocław Medical University, ul. Borowska 211a, 50-556 Wrocław, Poland; 2 Department of Biosystem Engineering and Chemical Processes, Opole University of Technology, ul. Stanisława Mikołajczyka 5, 45-271 Opole, Poland; s.czabaj@po.edu.pl; 3 Department of Fermentation and Cereals Technology, Wroclaw University of Environmental and Life Sciences, ul. Chełmońskiego 37, 51-630 Wrocław, Poland; 4 Department or Organic Chemistry, Faculty of Chemistry and Technology, University of Split, Ruđera Boškovića 35, 21000 Split, Croatia

**Keywords:** mead, honey wine, quality control, authenticity, liquid–liquid extraction

## Abstract

Volatile profiles of unifloral honeys and meads prepared in different ways (boiled-saturated, not boiled-unsaturated) were investigated by headspace solid-phase micro extraction (HS-SPME) and dehydration homogeneous liquid–liquid extraction (DHLLE) followed by GC-FID/MS analyses. The obtained data were analyzed by principal component analysis (PCA) to evaluate the differences between the investigated products. The volatile profiles of honey as well as the boiled and the not boiled meads prepared from it showed significant discrepancies. The meads contained more aliphatic acids and esters but fewer monoterpenes and aliphatic hydrocarbons than the honey. Significant/substantial differences were found between the boiled (more aliphatic alcohols and acids) and the not boiled meads (more aliphatic hydrocarbons and esters). Some compounds related to yeast metabolism, such as tryptophol, may be considered markers of honey fermentation. This research allowed us to identify chemical markers of botanical origin, retained and detectable in the meads: 4-isopropenylcyclohexa-1,3-diene-1-carboxylic acid and 4-(1-hydroxy-2-propanyl)cyclohexa-1,3-diene-1-carboxylic acid for linden; valeric acid, γ-valerolactone, *p*-hydroxybenzoic acid for buckwheat; 4-hydroxybenzeneacetic acid, homovanillic acid and *trans*-coniferyl alcohol for honeydew; and methyl syringate for canola.

## 1. Introduction

Mead (known also as honey wine) is a traditional alcoholic beverage prepared by fermentation of aqueous honey solution. It may be prepared using different honey varieties along with various additives and it is prepared in distinct conditions which have great impact on the final product characteristics [1]. Polish meads are registered and protected by the UE as Traditional Specialty Guaranteed. The traditional method of preparation involves gently boiling the wort (saturated mead), dissolving the honey in water, boiling at 95–105 °C and removing the coagulated impurities gathering on the top [2]. However, not boiled (unsaturated) meads are also available on the market and claim to preserve more of the original honey aroma. Besides different additives and modes of preparation, the honey variety shows the greatest impact on the final quality and aroma characteristics of the mead. This is particularly visible in traditional “show” mead, which is a plain mead containing, besides nutrient additives, only water and honey. Meads prepared from unifloral honeys may retain characteristic aroma properties and some of them, e.g., mead produced from linden honey, were particularly valued in the past [3]. However, the research literature seems to lack studies focusing on the volatile traceability markers in meads. During the preparation of the wort and during fermentation, different processes affect the chemical composition of the product. Heat treatment makes the fermentation process easier for yeast, thus accelerating the ethanol production, and gently boiled wort results in significantly higher antioxidant activity of the mead but also increases in HMF (5-hydroxymethylfurfural) level. Nevertheless, prolonged heating reduces the positive effects on antioxidant activity and total phenolic content of some honey types [1,4]. Therefore, according to Bednarek and Szwengiel, the HMF level and the phenolic compound profile are not good indicators of thermal processing of honey wort in commercial mead samples [5]. Still not much is known about the volatile composition of meads prepared from different unifloral honeys, as well as on differences between profiles of the boiled and not boiled meads. Just recently, Starowicz and Granvogl reported a study on the effect of wort boiling on the formation of volatiles and sensory properties of mead produced from multifloral honey, focusing on headspace volatiles [6]. The scope of this study was to: (i) investigate and, for the first time, compare volatile profiles of both headspace and liquid extracts of honey as well as boiled and not boiled meads from the most common unifloral varieties occurring in Poland (canola, buckwheat, linden, honeydew), (ii) investigate the changes in volatile profiles between honey and corresponding mead, (iii) evaluate the differences in volatile profiles between boiled and not boiled meads, for the first time include liquid extracts of volatiles, and select potential processing markers, (iv) determine the possible chemical marker compounds retained in the final product, which are useful to confirm the botanical origin of the honey used to prepare mead.

## 2. Results and Discussion

The compositions of four honey varieties and sugar syrup control along with corresponding samples of mead prepared in two different ways were investigated using gas chromatography mass spectrometry with a flame ionization detector (GC-FID/MS) following two complementary extraction methods, i.e., dehydration homogenous liquid–liquid extraction (DHLLE) and headspace solid phase micro extraction (HS-SPME). DHLLE is an extraction method based on addition of isopropanol to the aqueous sample and separation of the alcoholic extract by dehydration and centrifugation, followed by addition of dichloromethane, washing with water, drying and concentration [7,8]. Different extraction methods were selected in order to obtain more comprehensive profiles that include volatile and semi-volatile compounds. The performed analyses allowed us to track similarities and differences in volatile chemical profiles between original honey and meads prepared in different manners.

### 2.1. Headspace Profiles of the Investigated Mead and the Corresponding Honey Samples

The GC-FID/MS analyses of HS-SPME extracts allowed us to identify 115 compounds from different chemical groups in honey and mead (Table 1). In general, the major compound groups dominating the headspace profiles were monoterpenes, benzene derivatives and aliphatic compounds.

**Figure 1:**
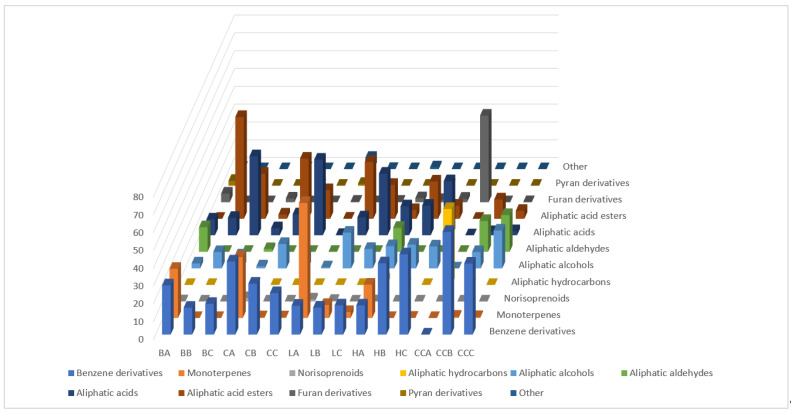
The overall percentages of the different volatile organic compound (VOC) structural groups in the analyzed honeys and meads by HS-SPME. BA, BB, BC—buckwheat honey, not boiled and boiled mead, respectively; CA, CB, CC—canola honey, not boiled and boiled mead, respectively; LA, LB, LC—lime tree honey, not boiled and boiled mead, respectively; HA, HB, HC—honeydew honey, not boiled and boiled mead, respectively; CCA, CCB, CCC— glucose–fructose syrup, not boiled and boiled mead, respectively.

**Figure 2:**
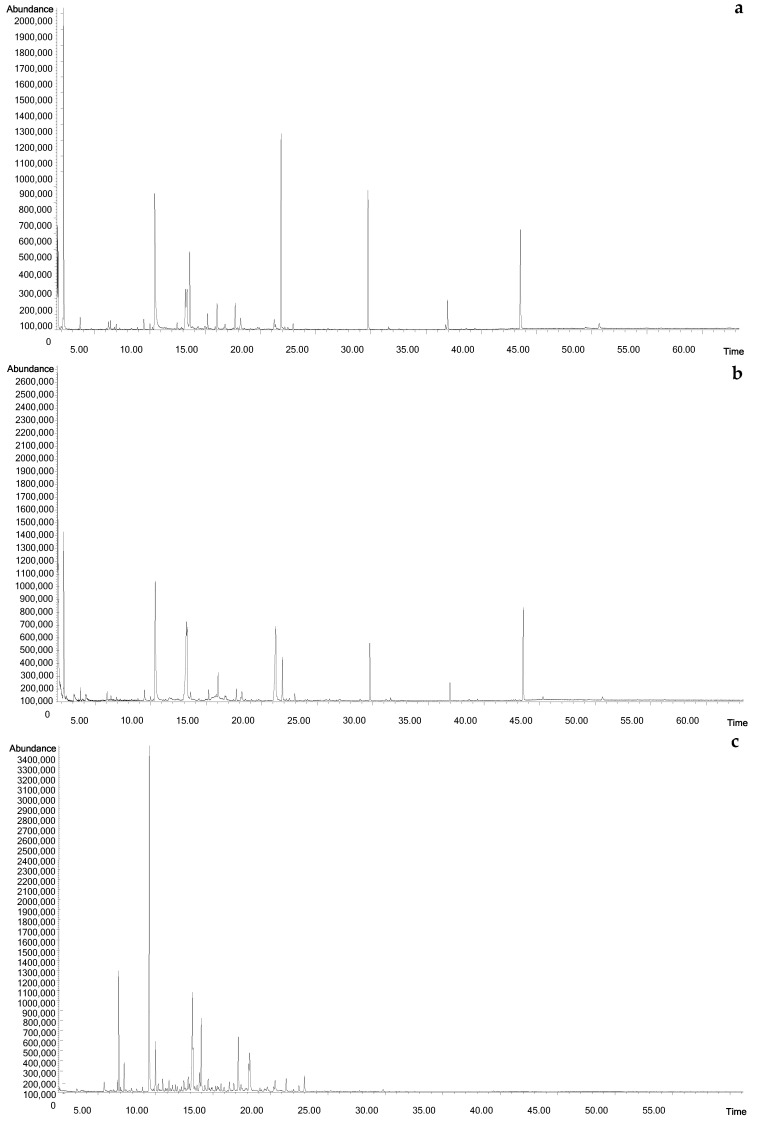
Comparison of the representative HS-SPME profiles of linden honey (**a**), not boiled mead (**b**) and boiled mead (**c**).

The comparison between headspace profiles of the meads and the corresponding unifloral honeys showed relevant differences (Figure 1 and Figure 2). The total percentage of highly volatile monoterpenes and norisoprenoids that were present in the honey dramatically decreased in the mead. Besides losses of VOCs together with CO_2_ during fermentation [9], an additional amount of volatiles was lost during heating, which resulted in an additional loss of monoterpenes in boiled meads. The total percentage of monoterpenes in the meads was reduced to zero or nearly zero (buckwheat, honeydew, canola) except for linden honey where it decreased by about 9- or 19-fold for not boiled and boiled mead, respectively. Higher preservation of monoterpenes in this variety in contrast to the other ones undoubtedly has an impact on the final, outstanding aroma. The levels of compounds most abundant in linden honey (*p*-cymenene—24.27%, *p*-cymen-8-ol—13.21% and 4-isopropylcyclohexa-1,3-dienecarbaldehyde—4.72%) significantly decreased in the meads. Only small aliquots of *p*-cymenene (0.72–0.80%) and *p*-cymen-8-ol (2.97%, only in the not boiled mead) were found, except for 4-isopropylcyclohexa-1,3-dienecarbaldehyde, which was not detected. This demonstrated that the not boiled meads retain a bit more of the original honey aroma. The total percentage of benzene derivatives reduced by about half in the case of buckwheat and canola meads, and remained stable in the case of linden, but increased threefold for the honeydew mead. Major compounds from this group present in the honeys were: benzaldehyde (13.11%; 27.34%; 0.90%; 6.64% in buckwheat, canola, linden and honeydew respectively), benzyl alcohol (3.02%; 1.61%; 1.65% in buckwheat, canola, honeydew, respectively), phenylacetaldehyde (5.11%; 2.19% in buckwheat and honeydew, respectively), 4-methylphenol in buckwheat (2.98%) and 2-phenylethanol in canola honey (4.13%).

After fermentation, the levels of major benzene derivatives notably decreased, except for 2-phenylethanol which was generated abundantly in all the honeys and sugar syrup control, reaching from 11.77 to 50.93%. After fermentation, in all honeys, 2-phenethyl acetate appeared (up to 4.25%) as well as other minor compounds. 2-Phenylathanol is the known fermentation product deriving from L-phenylalanine [10,11] and 2-phenethyl acetate characterizes fermentation by *Saccharomyces cerevisiae* yeast [12]. The fluctuations of their abundance in boiled and not boiled meads were very small. The fermentation process and the mode of preparation had strong impact on changes in amounts of different groups of aliphatic compounds in the headspace profiles. The levels of aliphatic hydrocarbons (0.49, 2.98, 42.61% for canola, honeydew, control, respectively) and aldehydes (13.82, 1.58, 13.46% for buckwheat, canola, honeydew, respectively) decreased to zero in the meads. The percentages of aliphatic alcohols, acids and esters significantly increased in the meads in comparison with floral honey types. The increase of aliphatic alcohols and esters was much higher for the not boiled meads than for the boiled ones. In the case of esters, the difference was 2-fold. The increase of aliphatic acids percentage was much higher in the boiled meads, from 2- to nearly 5-fold higher. The difference between percentage of aliphatic acids in honey and the not boiled mead depended on the honey type, i.e., for buckwheat and honeydew honey it was small and for canola and linden the difference was big. The meads were characterized by high total percentage of aliphatic esters synthetized during fermentation [13] reaching from 7.38% to 57.11% and 2–3 times more than in the corresponding honey. Interestingly, the not boiled meads contained notably more aliphatic esters than the boiled ones, which greatly affects the final aroma. A particularly high difference in ester percentage between the not boiled and the boiled honeys was related to the content of ethyl decanoate and ethyl dodecanoate in the not boiled (up to 23.91% and 13.43%, respectively) and in the boiled (up to 4.26% and 4.84%, respectively). Aliphatic esters are responsible for fruity odors [14]. Ethyl decanoate and ethyl dodecanoate are described, respectively, as sweet, fatty, nut-like, with a winey-cognac odor and oily, fatty, floral, with fatty fruity taste [13,15]. The percentage of pyran and furan derivatives were in general reduced in the meads compared to the corresponding honey, except for furan derivatives in the honeydew mead. The reduction of furans may be related to polymerization into melanoidins and their precipitation. 

### 2.2. DHLLE Extract Profiles of the Investigated Mead and the Corresponding Honey Samples

**Figure 3:**
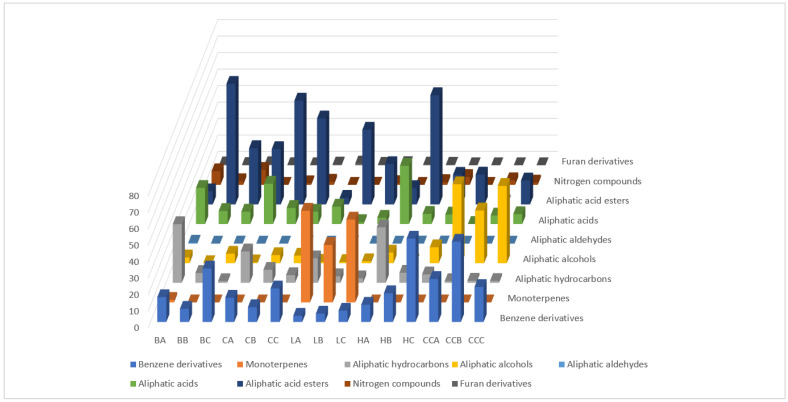
The overall percentages of the different volatile organic compound (VOC) structural groups in the analyzed honeys and meads by DHLLE. BA, BB, BC—buckwheat honey, not boiled and boiled mead, respectively; CA, CB, CC—canola honey, not boiled and boiled mead, respectively; LA, LB, LC—lime tree honey, not boiled and boiled mead, respectively; HA, HB, HC—honeydew honey, not boiled and boiled mead, respectively; CCA, CCB, CCC— glucose–fructose syrup, not boiled and boiled mead, respectively.

The GC-FID/MS analyses of the DHLLE extracts allowed us to identify 53 compounds from different chemical functional groups (Table 2). In contrast to the HS-SPME extracts, monoterpenes were relevant in the DHLLE extracts only for linden honey and meads. The major compound groups dominating the extracts were aliphatic compounds and benzene derivatives. Additionally, nitrogen compounds and furan derivatives were found in the extracts. The comparison between profiles of the meads and the corresponding unifloral honeys showed relevant differences (Figure 3). Within the group of aliphatic compounds, hydrocarbons dominated in the honeys and oxygenated compounds (mainly alcohols, acids and esters) were more abundant in the meads. There were observable trends in the abundance of aliphatic compounds, depending on mead preparation method. The not boiled meads contained more aliphatic hydrocarbons and esters while the boiled meads contained more aliphatic alcohols and acids. The most abundant hydrocarbon in honey was tricosane (up to 25.35%). The not boiled meads contained up to 5.20% of this compound and high percentage of esters, mainly ethyl hexadecanoate (up to 24.98%) and ethyl octadecanoate (up to 37.17%). The boiled meads contained lower percentages of these esters (up to 22.65% and 16.67%, respectively) but also contained higher amounts of acids and alcohols, mainly octadecanoic acid (up to 19.07%), hexadecanoic acid (up to 4.92%) and alcohols, mainly (*Z*)-octadec-9-en-1-ol (up to 3.81%) and octadecan-1-ol (up to. 4.54%). A similar trend was observed for benzene derivatives. The highest total percentage of these compounds was present in the boiled meads. In the not boiled meads it was higher, equal or lower than in the honey, depending on the botanical source. Substantial differences occurred after fermentation: 2-phenylethanol appeared in the meads, more abundant in the boiled (up to 17.11%) and less in the not boiled ones (up to 8.61%). In buckwheat mead, *p*-hydroxybenzoic acid level decreased in the not boiled (3.29%) and increased in the boiled (16.11%) meads in contrast to the honey (5.65%). On the other hand, the levels of 2,3-dihydrobenzofuran in the same honey type were 7.64%, 0.94% and 4.33% for the honey, the not boiled and the boiled mead, respectively. For the honeydew, the biggest differences were observed in homovanillic acid (0.36%, 0.35%, 4.89%) and coniferyl alcohol (0.47%, 1.68%, 3.46%) percentages observed in the honey, the not boiled and the boiled meads, respectively. A significant increase in the case of boiled meads was observed and could be explained by the hydrolysis of non-volatile derivatives of these compounds during cooking, for example. Nitrogen compounds were usually most abundant in the boiled meads. Tryptophol appeared only in the meads and was more abundant in the boiled (up to 4.81%) than in the not boiled ones (up to 1.68%). Buckwheat honey and meads also contained 1-isoquinolonecarbonitrile (1.37%, 0.49% 0.93%), 1*H*-indole-3-acetonitrile (3.58%, 0.02%, 1.38%) and 1*H*-indole-3-carboxaldehyde (3.30%, 0.78%, 5.00%). The latter was previously found in buckwheat honey [8] however detailed research is necessary to verify if these compounds may be considered chemical markers of buckwheat in the honey and mead. Monoterpenes were almost not detected except for linden honey and meads. The latter retained some part of 4-isopropenylcyclohexa-1,3-diene-1-carboxylic acid (22.83%, 15.05%, 23.64%) and 4-(1-hydroxy-2-propanyl)cyclohexa-1,3-diene-1-carboxylic acid (29.27%, 18.35%, 22.37%) in the honey and the not boiled or the boiled mead, respectively. Interestingly, the not boiled meads retained less of them. DHLLE extracts allowed us also to detect some furan compounds: small amounts of 2,4-dihydroxy-2,5-dimethyl-3(2H)-furan-3-one in all the honey and mead samples and γ-valerolactone in the buckwheat honey and meads.

### 2.3. Traceability of Chemical Markers of Botanical Origin and Comparison with Control

The comparison of volatile profiles of honey and meads allowed us to detect characteristic compounds that could be helpful to trace botanical origins of the honey used to produce mead and which are detectable after fermentation. In most of the samples, DHLLE extracts were found to be more useful to find such characteristic compounds. This is also related to the fact that it focuses on the less volatile compounds. In contrast, in the case of HS-SPME the main targets are the more volatile compounds present in the headspace that are partially lost during wort preparation and fermentation process. Pentanoic (valeric) acid, previously reported as a volatile marker in the headspace of buckwheat honey [16], in the current study was detected by HS-SPME in the honey and not boiled mead (0.70; 0.20%). DHLLE extracts provided a wider range of characteristic compounds for buckwheat honey: *p*-hydroxybenzoic acid (5.65; 3.29; 16.11%), 1-isoquinolinecarbonitrile (1.37; 0.49; 0.93%), 1*H*-indole-3-acetonitrile (3.58; 0.02; 1.38%), 1*H*-indole-3-carboxaldehyde (3.58; 0.02; 1.38%) and γ-valerolactone (0.14; 0.04; 0.29%) were detected in the honey and both meads, respectively. The compounds were previously found in chromatographic profiles of this honey type and *p*-hydroxybenzoic acid was proposed as its potential marker [8,17]. In canola honey, the compound that may be useful as a nonspecific marker and that was retained in both honey and meads was methyl syringate (10.55; 4.45; 9.73%). This lignin derivative is also present, however, in a number of other honey varieties [18]. DHLLE extracts retained particularly high percentages of less volatile terpenic acids: 4-isopropenylcyclohexa-1,3-diene-1-carboxylic acid (22.83; 15.05; 23.64%) and 4-(2-hydroxy-2-propanyl)cyclohexa-1,3-diene-1-carboxylic acid (29.27; 18.35; 22.37%). These compounds were previously reported as a markers of linden honey found in its SPE extracts [19]. Interestingly, the percentage of these compounds was higher in the boiled meads than in the not boiled ones. This could be related to the hydrolysis of glycosidic precursors of these compounds that were also found in linden honey during cooking [20]. In DHLLE extracts of honeydew honey and mead, 4-hydroxybenzeneacetic acid (*p*-hydroxyphenylacetic acid) (6.40; 0.84; 6.94%), homovanillic acid (0.36; 0.35; 4.89%) and *trans*-coniferyl alcohol (0.47; 1.68; 3.46%) were found. According to Isidorov et al., 4-hydroxybenzeneacetic and homovanillic acids are characteristic of honeydew honey [21]. *p*-Hydroxyphenylacetic acid and coniferyl alcohol were previously found in fir honeydew honey and the latter was proposed as its potential marker [22].

In comparison with the meads, the fermented glucose–fructose syrup did not contain any of the characteristic compounds that could be attributed to one of the analyzed honey types and were characterized by quite poor volatile profiles. The headspace of such fermented products (not boiled and boiled, respectively) was dominated by 2-phenylethanol (50.93; 31.98%), 2-methylpropan-1-ol (8.83; 20.65%) and 3-methylbutanal (17.32; 20.65%) that appeared after fermentation. The DHLLE extract contained characteristic products of the yeast fermentation (not boiled and boiled, respectively): 2-phenylethanol (25.32; 8.07%) and tryptophol (2.79; 1.38%), but also relevant amounts of aliphatic alcohols that were already present in the glucose–fructose syrup (syrup, not boiled and boiled fermented syrup, respectively): hexadecan-1-ol (3.40; 0.28; 3.33%), (*Z*)-octadec-9-en-1-ol (30.46; 18.90; 30.42%), octadecan-1-ol (13.97; 12.74; 13.09%). Moreover, the extracts contained remarkable percentages of phthalates and tributyl acetylcitrate, probably originating from contact with plastic packaging.

### 2.4. Comparison between Volatile Profiles of Honey, Boiled and Not Boiled Meads–Principal Component Analysis (PCA)

General observation of the volatile profiles demonstrated that the meads contain fewer terpene compounds than honey, but more aliphatic compounds. Depending on the method of preparation, different groups of compounds dominate the final product. Aliphatic acids are more abundant in the boiled meads and more aliphatic esters are present in the not boiled meads. As a result of fermentation, a number of compounds appear in the mead. Among them are mostly esters (DHLLE): 2-phenethyl acetate, ethyl acetate, isoamyl acetate, ethyl octanoate, ethyl decanoate, ethyl dodecanoate, ethyl tetradecanoate, ethyl hexadecanoate (HS-SPME), ethyl decanoate, ethyl dodecanoate, ethyl tetradecanoate, ethyl hexadec-9-enoate, ethyl hexadecanoate, ethyl octadecanoate (DHLLE). The findings were similar for aliphatic acids: octanoic acid and decanoic acid (particularly abundant in boiled meads), acetic acid (HS-SPME), and octanoic acid. On the other hand, octadecanoic acid was most abundant in the analyzed honeys and its percentage was usually higher in the not boiled meads in comparison to the boiled ones. Other relevant differences were increases of 2-phenylethanol and 3-methylbutan-1-ol level (HS-SPME), increases of 2-phenylethanol and appearance of tryptophol (DHLLE) in the meads. 2-Phenylethanol and tryptophol are known to derive, respectively, from phenylalanine and tryptophan as *Saccharomyces cerevisiae* catabolism products [23]. The esters are formed as a result of yeast fermentation and have high impact on the aroma of alcoholic beverages, providing fruity notes. The ester formation depends on different factors, but particularly on the concentration of nitrogen compounds and must solids [24,25,26]. Since preparation of boiled meads involves cooking and removal of solid impurities together with coagulated proteins before fermentation, it may be related with lower amounts of ester compounds in the final product.

**Figure 4:**
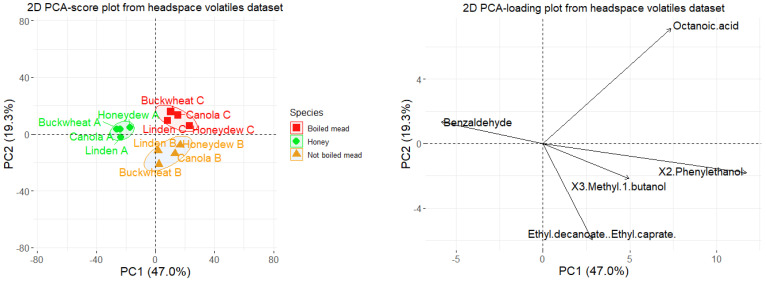
Principal component analysis (PCA) scores (**left**) and loadings (**right**, only first five variables with the highest contributions are shown) plots based on the HS-SPME volatiles extract dataset.

**Figure 5:**
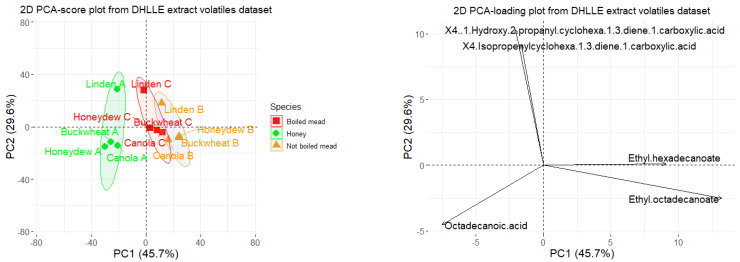
Principal component analysis (PCA) scores (**left**) and loadings (**right**, only first five variables with the highest contributions are shown) plots based on the DHLLE volatiles extract dataset.

The datasets containing volatile compositions of honey and meads extracts obtained by DHLLE and HS-SPME were subjected to PCA analysis after mean-centering of the data (Figure 4 and Figure 5). In the case of the data obtained for headspace volatiles, the first two factors explained 66.3% (and three 80.1%) of the variance among the samples and in the case of the data obtained for solvent extracts 75.3% (and three 85.5%). In both cases, natural clustering of honey, boiled and not boiled meads was observed regardless of the botanical origin. The variables providing the greatest contribution to the principal components and differentiation of the groups of samples, based on headspace, were: 2-phenylethanol, octanoic acid, benzaldehyde, 3-methylbutan-1-ol (accounting about 43%, 17%, 11% and 8% of PC1), octanoic acid, ethyl decanoate, decanoic acid, ethyl dodecanoate, 3-methylbutan-1-ol (accounting about 39%, 27%, 13%, 7% and 3% of PC2).

The variables providing the greatest contribution to the principal components and differentiation of the groups of samples, based on DHLLE extracts, were: ethyl octadecanoate, ethyl hexadecanoate, octadecanoic acid, tricosane (accounting about 45%, 21%, 15% and 13% of PC1). In case of PC2, it was responsible for distinguishing between samples of linden origin from other samples and the major variables accounting for about 66% of PC2 in total were compounds characteristic for linden honey: 4-isopropenylcyclohexa-1,3-diene-1-carboxylic acid and 4-(2-hydroxy-2-propanyl)cyclohexa-1,3-diene-1-carboxylic acid. The samples naturally formed three groups. In the case of DHLLE extracts, octadecanoic acid characterized honeys and ethyl hexadecanoate and ethyl octadecanoate characterized meads.

## 3. Materials and Methods

### 3.1. Materials and Samples

Analytical grade chemicals, isopropanol, ethanol, dichloromethane, anhydrous MgSO_4_, and Na_2_SO_4_, were obtained from Chempur (Piekary Śląskie, Poland). Samples of different varietal honeys were provided by Miody Polskie Sp. z o.o. (Mokra, Poland). The certified honey samples were obtained from professional beekeepers in Poland. The honey samples were stored at 4 °C in glass jars, in the dark. For wort preparation, the following varieties were used: buckwheat (*Fagopyrum esculentum* Moench), canola (rapeseed) (*Brassica napus* L.) and honeydew. Glucose–fructose syrup from Cargill Poland (Warszawa, Poland) was used as control. The samples of meads were prepared by fermentation of mead wort at room temperature (18–20 °C) with *Saccharomyces cerevisiae* commercial strain, Safspirit FD-3, Fermentis (Lesaffre, France). Dry yeast obtained from its manufacturer was rehydrated for 30 min in sterile water before inoculation of worts (36 °Bx) prepared from honey and water (1:2; *v*/*v*) in two different modes and supplemented with addition of diammonium phosphate (DAP) as a nutrient (0.4 g/L). Boiled meads were prepared by gentle boiling of the honey wort for 30 min, with removal of foam gathering on the top and replenishment of evaporated water. Not boiled meads were prepared by fermentation of honey wort prepared without heat treatment. The fermentation was continued until the daily loss of weight (in terms of CO_2_ loss) was lower than 0.5 g, which was an indication of fermentation termination.

### 3.2. Headspace Solid-Phase Microextraction (HS-SPME)

The headspace volatiles were extracted using a polydimethylsiloxane/divinylbenzene (PDMS/DVB) fiber in manual SPME holder, conditioned according to the manufacturer’s (Supelco (Bellefonte, PA, USA)) instructions. The honey samples were dissolved in water solution saturated with NaCl (5 mL, 1:1 (*v*/*v*)); the mead samples were saturated with NaCl and placed in a 15 mL glass vial sealed with polytetrafluorethylene (PTFE)/silicone septa. The vial was conditioned at 60 °C in a water bath; after 15 min of conditioning the headspace volatiles were extracted for 45 min under constant stirring (1000 rpm) of the solution with a magnetic stirrer. After sampling, the SPME fiber was inserted into the injector (250 °C) of the GC-FID and GC-MS for 6 min to perform analyses.

### 3.3. Dehydration Homogenous Liquid–Liquid Extraction Method

The sample preparation was performed similarly as reported previously [7] with slight modifications. In short, in the case of honey, the solution made of 5 g of honey dissolved in 6 mL of ultrapure water was placed in 15 mL centrifuge tube. Afterwards, 2 mL of an isopropanol-ethanol mixture (1:1, *v*/*v*) was added to the solution. In the case of meads, to 10 mL of mead, 1 mL of isopropanol was added. Afterwards, 6 g of MgSO_4_ was gradually added and carefully mixed to dehydrate the sample placed in a cold water bath. The tubes were centrifuged (5 min, 3000 rpm) to provide separation of the two phases obtained. The upper layer containing alcoholic extract was transferred to another probe tube and diluted with 1 mL of dichloromethane. In the next step, it was washed consecutively with three 1 mL portions of ultrapure water, dried using anhydrous Na_2_SO_4_ and carefully concentrated under Vigreaux column. Then, 4 µL of the extract was used for GC-FID/MS analyses.

### 3.4. Chromatographic Conditions

For the GC-FID analyses, a 7890A gas chromatograph coupled to FID detector (Agilent Technologies, Palo Alto, CA, USA) and HP-5MS capillary column (5% phenyl-methylpolysiloxane, 30 m, 0.25 mm i.d., coating 0.25 μm, Agilent) were applied. The GC conditions were set as previously described [7]. The oven temperature was set as isothermal at 70 °C for 2 min; the temperature increased from 70 to 200 °C by 3 °C·min^−1^ and afterwards was held isothermal at 200 °C for another 15 min. The carrier gas used was He (1.0 mL·min^−1^). The temperatures of injector and the FID detector were set to 250 °C and 300 °C, respectively. The GC-MS analyses were done using a similar gas chromatograph coupled to a mass selective detector (MSD) model 5977E (Agilent Technologies, Palo Alto, CA, USA) and the same chromatographic settings as for the GC-FID analyses. The MSD worked in in EI mode (70 eV) in the mass range 30–300 amu and the ion source temperature was 230 °C. The identification of the volatile organic compounds (VOCs) involved comparison of their retention indices (RI) calculated based on C_9_-C_25_ *n*-alkanes retention times with those reported in the literature [27] and their mass spectra with those of available authentic compounds or listed in mass spectral libraries: Wiley 9 (Wiley, New York, NY, USA) and/or NIST 14 (Gaithersburg, MD, USA). The percentage composition of VOCs was calculated from the GC peak areas without correction factors, using normalization method.

### 3.5. Statistics

The obtained data were evaluated by principal component analysis (PCA) after mean-centering. The analyses were performed using R for Windows, version 4.0.0 (R-Cran project, http://cran.r-project.org/ accessed on 7 June 2022) including the “factoextra” library [28].

## 4. Conclusions

The comparison of HS-SPME and DHLLE volatile profiles allowed us to determine differences between composition of honey (containing more monoterpenes and aliphatic hydrocarbons), boiled (containing more aliphatic alcohols and acids) and not boiled meads (containing more aliphatic esters) and to observe variety-specific marker compounds in the honey-derived products. The chemical profiles obtained using different preparation techniques provided partially distinct data; thus, the research demonstrates that HS-SPME and DHLLE may be used as complementary techniques. Both methods may be useful to determine the origin of honey used to prepare mead, as well as to verify the mode of its preparation involving heat treatment (boiled) or lack of (not boiled) in terms of quality control based on the selected marker compounds characteristic for botanical origin (e.g., 4-isopropenylcyclohexa-1,3-diene-1-carboxylic acid and 4-(1-hydroxy-2-propanyl)cyclohexa-1,3-diene-1-carboxylic acid for linden; valeric acid, γ-valerolactone, *p*-hydroxybenzoic acid for buckwheat; 4-hydroxybenzeneacetic acid, homovanillic acid and *trans*-coniferyl alcohol for honeydew; and methyl syringate for canola) or processing (e.g., benzaldehyde for honey; octanoic acid for boiled mead; 2-phenylethanol, ethyl caprate, ethyl hexadecanoate, ethyl octadecanoate and 3-methylbutan-1-ol for not boiled mead).

## Figures and Tables

**Table 1:** Comparison of the HS-SPME profiles of meads and related unifloral honeys.

No.	Compound	RI ^1^	Buckwheat			Canola			Linden			Honeydew			Control		
			A	B	C	A	B	C	A	B	C	A	B	C	A	B	C
			Area [%]														
	***Benzene derivatives***																
1	Isopropylbenzene	932	-	-	-	-	-	-	0.20	0.15	0.18	-	-	-	-	-	-
2	Benzaldehyde	967	13.11	0.10	0.10	27.34	-	-	0.90	0.05	-	6.64	0.34	0.16	-	0.93	2.11
3	2-Phenylpropene	987	-	-	-	-	-	-	-	0.08	-	-	-	-	-	-	-
4	Benzyl alcohol	1043	3.02	-	-	1.61	0.44	0.20	-	0.06	-	1.65	0.05	0.02	-	-	-
5	Phenylacetaldehyde	1049	5.11	-	-	1.09	-	-	0.28	-	-	2.19	0.25	0.15	-	0.34	0.36
6	Acetophenone (1-Phenylethanone)	1072	-	-	-	-	-	-	-	-	-	-	0.13	0.23	-	-	-
7	4-Methylphenol	1086	2.98	-	-	-	-	-	-	-	-	-	-	-	-	-	-
8	2-Phenylethanol	1120	0.02	13.37	11.77	4.13	26.57	20.94	0.17	12.73	13.40	0.70	35.13	38.32	-	50.93	31.98
9	Ethyl benzoate	1175	-	-	-	1.48	-	-	-	-	-	-	-	-	-	-	-
10	4,5,6,7-Tetrahydro-3,6-dimethylbenzofuran	1176	-	-	-	-	-	-	0.60	-	-	-	-	-	-	-	-
11	Benzoic acid	1185	-	-	-	1.34	-	-	-	-	-	-	-	-	-	-	-
12	Methyl salicylate	1196	-	-	-	-	-	-	-	-	-	0.85	-	-	-	-	-
13	2-(4′-Methylphenyl)propanal	1207	-	-	-	-	-	-	6.05	-	-	-	-	-	-	-	-
14	4,7-Dimethylbenzofuran	1216	-	-	-	-	-	-	0.56	-	-	-	-	-	-	-	-
15	Ethyl phenylacetate	1248	-	-	-	1.62	-	-	-	-	-	-	0.24	0.10	-	-	-
16	2-Phenethyl acetate	1260	-	1.59	4.14	-	1.31	1.61	-	1.74	2.31	-	3.01	4.25	-	-	-
17	2-Phenylbut-2-enal *	1268	-	-	-	-	-	-	0.96	-	-	-	-	-	-	-	-
18	4-Propylbenzaldehyde *	1275	-	-	0.72	-	-	-	-	-	-	-	0.41	0.70	-	1.88	1.07
19	1-(2-Aminophenyl)ethanone	1303	0.68	-	-	-	-	-	-	-	-	-	-	-	-	-	-
20	4-Vinyl-2-methoxyphenol	1317	-	-	-	-	0.03	-	-	-	0.29	-	-	-	-	-	-
21	Ethyl benzenepropanoate	1351	-	-	-	-	0.20	0.33	-	-	-	-	-	-	-	-	-
22	3,5-Dimethoxybenzaldehyde (Syringaldehyde)	1440	-	-	-	1.07	-	-	-	-	-	-	-	-	-	-	-
23	2,4-Bis(1,1-dimethylethyl)phenol (BHT)	1518	-	-	0.22	-	-	-	-	0.05	0.08	-	0.12	0.42	-	1.58	1.25
24	Diisobutyl phthalate	1896	2.06	-	0.28	1.01	0.03	-	0.09	0.13	0.11	2.49	0.32	0.51	-	1.42	2.23
25	Dibutyl phthalate	1962	0.62	-	-	0.34	-	0.20	-	-	-	0.95	-	0.19	-	0.56	0.82
	***Monoterpenes***																
26	2,3-Dimethylbicyclo [2.2.1]hept-2-ene (Santene)	<900	-	-	-	-	-	-	-	-	-	0.69	-	-	-	-	-
27	α-Pinene	941	-	-	-	-	-	-	-	-	-	0.45	-	-	-	-	-
28	(*E*)-3,3-Dimethyl-δ1,α-cyclohexaneacetaldehyde *	1013	-	-	-	-	-	-	6.37	0.12	-	0.84	-	-	-	-	-
29	*p*-Cymene	1029	-	-	-	0.30	-	-	1.74	0.10	0.28	0.26	-	-	-	0.15	0.05
30	Limonene	1034	-	-	-	-	-	-	-	-	-	-	-	-	-	0.20	0.11
31	1,8-Cineole	1037	-	-	-	-	-	-	-	-	-	0.93	-	-	-	-	-
32	γ-Terpinene	1063	-	-	-	-	0.33	0.20	-	-	-	-	-	-	-	-	-
33	*trans*-Linalool oxide	1077	5.52	-	-	5.86	-	-	-	0.13	0.32	1.54	-	-	-	-	-
34	*cis*-Linalool oxide	1091	3.51	-	-	5.17	-	-	-	-	-	0.21	-	-	-	-	-
35	1-Methyl-4-(1-methylethenyl)-benzene (*p*-Cymenene)	1092	-	-	-	-	-	-	24.27	0.73	0.80	-	-	0.16	-	0.38	0.38
36	Linalool	1102	-	-	-	0.47	-	-	0.25	-	-	3.80	-	-	-	-	-
37	Hotrienol	1106	9.79	-	-	14.15	0.11	0.20	3.91	0.39	0.39	-	-	-	-	-	-
38	*trans-p*-Mentha-2,8-dien-1-ol	1125	-	-	-	-	-	-	1.10	-	-	-	-	-	-	-	-
39	*cis-p*-Mentha-2,8-dien-1-ol	1140	-	-	-	-	-	-	0.94	0.23	0.10	-	-	-	-	-	-
40	Lilac aldehyde A	1146	-	-	-	2.12	-	-	-	-	-	-	-	-	-	-	-
41	1-(1,4-Dimethyl-3-cyclohexen-1-yl)ethanone	1154	-	-	-	-	-	-	0.62	-	-	-	-	-	-	-	-
42	Lilac aldehyde B	1155	1.43	-	-	4.20	-	-	-	-	-	-	-	-	-	-	-
43	Menthofuran	1167	-	-	-	-	-	-	0.41	-	-	-	-	-	-	-	-
44	Lilac aldehyde D	1169	1.43	-	-	1.60	-	-	-	-	-	-	-	-	-	-	-
45	Borneol	1172	0.60	-	-	-	-	-	1.06	0.42	-	2.01	-	-	-	-	-
46	Terpinen-4-ol	1181	-	-	-	-	-	-	-	0.35	0.10	1.38	-	-	-	-	-
47	*trans*-Car-2-en-4-ol *	1184	-	-	-	-	-	-	0.80	-	-	-	-	-	-	-	-
48	*p*-Cymen-8-ol	1189	2.07	-	-	-	-	-	13.21	2.97	-	1.73	-	-	-	-	-
49	α-Terpineol	1194	-	-	-	-	-	-	-	-	-	5.71	-	-	-	-	-
50	*trans*-Isopiperitenol	1204	-	-	-	-	-	-	2.10	-	-	-	-	-	-	-	-
51	*p*-Menth-1-en-9-al (isomer I)	1218	3.24	-	-	0.31	-	-	-	-	-	-	-	-	-	-	-
52	*cis*-Isopiperitenol	1223	-	-	-	-	-	-	1.29	-	-	-	-	-	-	-	-
53	*trans-*Carveol	1224	-	-	-	-	-	-	-	0.29	-	-	-	-	-	-	-
54	4-(1-Methylethyl)benzaldehyde (Cuminal)	1243	-	-	-	-	-	-	0.49	-	-	-	-	-	-	-	-
55	Carvotanacetone	1251	-	-	-	-	-	-	0.70	-	-	-	-	-	-	-	-
56	Piperitone	1258	-	-	-	-	-	-	0.42	-	-	-	-	-	-	-	-
57	4-Isopropylcyclohexa-1,3-dienecarbaldehyde	1286	-	-	-	-	-	-	4.72	-	-	-	-	-	-	-	-
58	2-Methyl-3-phenylprop-2-enal *	1292	-	-	-	-	-	-	0.64	-	-	-	-	-	-	-	-
59	Thymol	1293	-	-	-	-	-	-	0.65	0.22	-	-	-	-	-	-	-
60	Carvacrol	1308	-	-	-	-	-	-	4.32	0.89	1.04	-	-	-	-	-	-
61	Limonene-1.2-diol	1347	-	-	-	-	-	-	0.84	0.59	0.35	-	-	-	-	-	-
	***Norisoprenoids***																
62	Isophorone	1126	-	-	-	0.93	-	-	-	-	-	-	-	-	-	-	-
63	4-Ketoisophorone	1148	0.09	-	-	-	-	-	-	-	-	0.11	-	-	-	-	-
64	*trans*-β-Damascenone	1385	-	-	-	0.98	-	-	1.13	0.33	-	-	-	-	-	-	-
	***Aliphatic hydrocarbons***																
65	Octane	<900	-	-	-	0.49	-	-	-	-	-	2.98	-	-	42.61	-	-
	***Aliphatic alcohols***																
66	2-Methylpropan-1-ol	<900	-	-	-	-	-	-	-	3.41	-	-	-	0.49	-	8.83	20.65
67	3-Methylbutan-1-ol	<900	0.92	9.15	7.26	0.26	13.70	8.06	-	16.64	10.93	0.64	13.31	11.83	-	-	-
68	2-Methylbutan-1-ol	<900	1.87	-	-	-	-	-	-	-	-	-	-	-	-	-	-
69	Heptan-2-ol	902	-	-	-	-	-	-	-	-	-	1.34	-	-	-	-	-
70	2-Ethylhexan-1-ol	1034	-	-	-	0.89	-	-	-	-	-	2.12	-	-	-	-	-
71	Octan-1-ol	1076	-	-	-	-	-	-	0.61	-	-	1.69	-	-	-	-	-
72	Nonan-1-ol	1177	-	-	-	-	-	-	-	-	-	6.10	-	-	-	-	-
73	Dodecan-1-ol	1478	-	-	-	-	-	-	-	-	-	0.59	-	-	-	0.36	0.60
74	***Aliphatic aldehydes***																
	3-Methylbutanal	<900	7.98	-	-	0.19	-	-	-	-	-	0.64	-	-	-	17.32	20.65
75	2-Methylbutanal	<900	5.20	-	-	-	-	-	-	-	-	-	-	-	-	-	-
76	Pentanal	<900	0.64	-	-	-	-	-	-	-	-	-	-	-	-	-	-
77	3-Methylpentanal	<900	-	-	-	1.39	-	-	-	-	-	-	-	-	-	-	-
78	Octanal	1004	-	-	-	-	-	-	-	-	-	1.74	-	-	-	-	-
79	Nonanal	1105	-	-	-	-	-	-	-	-	-	9.75	-	-	-	-	-
80	Decanal	1206	-	-	-	-	-	-	-	-	-	1.33	-	-	-	-	-
	***Aliphatic acids***																
81	Acetic acid	<900	-	6.46	0.10	0.19	-	-	-	4.02	-	0.33	4.83	0.10	-	-	-
82	Butanoic acid	<900	0.40	-	-	-	-	-	-	-	-	-	-	-	-	-	-
83	Pentanoic acid (Valeric acid)	<900	0.70	0.20	-	-	-	-	-	-	-	-	-	-	-	-	-
84	3-Methylpentanoic acid (3-Methylvaleric acid)	953	-	-	-	0.72	-	-	-	-	-	-	-	-	-	-	-
85	Hexanoic acid	987	1.30	1.00	1.88	0.36	1.86	1.90	0.15	0.65	0.96	0.64	0.58	0.69	-	-	-
86	2-Ethylhexanoic acid	1132	0.85	-	-	0.76			-	-	-	1.46	-	-	-	-	-
87	Octanoic acid	1185	2.66	2.03	29.14	1.17	9.61	28.01	0.05	4.49	20.15	6.40	8.74	20.47	-	0.90	1.16
88	Nonanoic acid	1283	2.77	-	-	0.68			-	-	-	6.73	-	-	-	-	-
89	Decanoic acid	1379	0.21	0.10	12.96	0.10	0.31	12.55	-	0.92	13.21	0.71	2.51	9.09	-	0.92	1.17
90	Dodecanoic acid	1573	-	-	-	-	-	-	-	-	0.27	-	-	-	-	-	0.00
91	Hexadecanoic acid	1967	-	-	0.30	-	-	-	-	-	-	-	-	-	-	-	-
	***Aliphatic acid esters***																
92	Ethyl acetate	<900	-	5.08	1.91	-	-	-	-	2.45	-	1.34	5.03	1.77	-	-	-
93	Ethyl butanoate	<900	-	-	-	-	0.11	0.10	-	-	-	-	0.09	0.08	-	-	-
94	Isoamyl acetate	<900	-	3.10	2.57	-	0.66	1.30	-	0.58	0.61	-	-	-	-	-	-
95	Ethyl hexanoate	999	-	0.50	0.92	0.17	1.53	1.10	0.08	0.47	0.93	0.54	0.78	1.19	-	0.55	0.72
96	Ethyl octanoate (Ethyl caprylate)	1198	-	4.86	1.99	0.14	2.83	1.04	-	4.36	0.77	-	4.97	1.42	-	4.75	0.64
97	Ethyl decanoate (Ethyl caprate)	1396	-	23.91	4.26	-	17.34	3.41	-	10.22	3.40	-	5.70	1.55	-	3.06	0.82
98	Ethyl dodecanoate	1595	-	13.43	2.74	-	10.08	4.24	-	7.59	4.84	-	1.63	0.25	-	1.64	0.85
99	2-Methyl-1-(1,1-dimethylethyl)-2-methylpropanoic acid, 1,3-propanediyl ester	1596	-	-	-	2.07	-	-	-	-	-	3.04	-	0.36	-	-	-
100	Isopentyl decanoate	1646	-	-	-	-	-	0.28	-	0.08	0.32	-	-	-	-	-	-
101	Diethyl decanedioate (Diethyl sebacate)	1789	-	0.30	-	-	-	-	-	-	-	-	-	-	-	-	-
102	Ethyl tetradecanoate (Ethyl myristate)	1794	-	1.25	1.40	-	0.05	0.82	-	-	-	-	0.28	0.11	-	0.21	0.35
103	Ethyl hexadec-9-enoate	1973	-	-	0.37	-	-	-	-	-	-	-	-	-	-	-	-
104	Ethyl hexadecanoate (Ethyl palmitate)	1993	-	4.68	9.11	-	1.00	3.80	-	5.81	7.65	-	2.43	0.65	-	0.63	1.07
105	Ethyl octadecanoate	2194	-	-	-	-	0.15	-	-	0.37	0.51	-	-	-	-	-	-
	***Furan derivatives***																
106	2-Methylfuran	<900	-	-	-	-	-	-	-	-	-	-	-	-	48.57	-	-
107	2,5-Dimethylfuran	<900	-	-	-	-	-	-	-	-	-	0.31	-	-	-	-	-
108	2-Furancarboxaldehyde	<900	2.07	-	-	2.06	-	-	0.31	-	-	1.30	1.27	1.97	-	-	0.05
109	2-Acetylfuran	916	2.81	-	-	-	-	-	-	-	-	0.62	-	-	-	-	-
110	5-Hydroxymethylfurfural	1247	-	-	-	-	-	-	-	-	0.32	-	-	-	-	-	-
	***Pyran derivatives***																
111	δ-Valerolactone	961	2.72	-	-	-	-	-	-	-	-	-	-	-	-	-	-
112	*cis*-Rose oxide	1114	-	-	-	-	-	-	0.72	-	-	-	-	-	-	-	-
113	*trans*-Rose oxide	1131	-	-	-	-	-	-	0.31	-	-	-	-	-	-	-	-
	***Other***																
114	Dimethyl disulfide	<900	0.81	-	-	0.43	-	-	-	-	-	-	-	-	-	-	-
115	Nonan-2-one	1094	-	-	-	-	-	-	-	-	-	1.01	-	-	-	-	-

A—honey, B—not boiled mead, C—boiled mead; ^1^ RI: retention indices determined relative to *n*-alkanes (C_9_-C_25_) on the HP-5MS column; *—tentatively identified; names of compound groups were marked in bold/italic.

**Table 2:** Comparison of the DHLLE extract profiles of meads and related unifloral honeys.

Compound		RI ^1^	Buckwheat			Canola			Linden			Honeydew			Control		
			A	B	C	A	B	C	A	B	C	A	B	C	A	B	C
			Area [%]														
***Benzene derivatives***																	
1	2-Phenylethanol	1116	-	1.95	4.06	-	1.56	3.70	-	2.37	1.79	-	8.61	17.11	-	25.32	8.07
2	Benzoic acid	1162	-	-	-	-	0.07	-	-	-	-	0.40	-	-	-	-	-
3	2,3-Dihydrobenzofuran (Coumaran)	1249	7.64	0.94	4.33	0.63	0.03	0.77	0.50	0.10	0.09	0.23		0.53	-	-	-
4	Phenylacetic acid	1269	-	-	-	-	-	0.19	-	-	-	-	-	-	-	-	-
5	4-Vinyl-2-methoxyphenol	1314	0.32	-	0.32	1.18	0.05	0.79	0.64	0.10	0.39	0.53	0.16	0.95	-	-	-
6	4-Hydroxyphenethyl alcohol	1445		0.35	1.23		0.09	0.35	0.91	0.73	3.04		4.42	9.11	-	-	-
7	2,4-Bis(1,1-dimethylethyl)phenol (BHT)	1517	0.91	1.04	2.77	1.65	1.72	2.92	0.58	1.12	0.53	1.34	0.95	4.79	0.26	6.33	4.52
8	*p*-Hydroxybenzoic acid	1575	5.65	3.29	16.11	-	-	-	-	-	-	-	-	-	-	-	-
9	4-Hydroxybenzeneacetic acid *	1608	-	-	-	-	-	-	-	-	-	6.40	0.84	6.94	-	-	-
10	Homovanillic acid	1657	-	-	-	-	-	-				0.36	0.35	4.89	-	-	-
11	*trans*-Coniferyl alcohol	1744	-	-	-	-	-	-	-	-	-	0.47	1.68	3.46	-	-	-
12	Methyl syringate (Methyl 4-hydroxy-3,5-dimethoxybenzoate)	1774	0.48	0.01	0.77	10.55	4.45	9.73	0.87	0.35	0.68	-	-	-	-	-	-
13	*p*-Coumaric acid	1849	0.02	-	0.81	-	-	-	-	-	-	-	-	-	-	-	-
14	Diisobutyl phthalate	1869	-	0.09	0.87	-	0.32	0.57	-	0.05	0.12	-	0.12	0.50	6.90	5.11	2.61
15	Dibutyl phthalate	1962	-	0.28	1.13	0.67	0.66	1.31	0.24	0.24	0.18	0.71	0.29	2.21	18.91	11.87	5.99
	***Monoterpenes***																
16	*p*-Mentha-1,5,8-triene	1030	-	-	-	-	-	-	0.08	-	-	-	-	-	-	-	-
17	*p*-Cymenene	1092	-	-	-	-	-	-	0.37		0.11	-	-	-	-	-	-
18	Limonen-1,2-diol	1348	-	-	-	-	-	-	0.37	0.10	0.24	-	-	-	-	-	-
19	4-(1-Methylethyl)benzoic acid (Cumic acid)	1437	-	-	-	-	-	-	2.62	1.27	3.83	-	-	-	-	-	-
20	4-Isopropenylcyclohexa-1,3-diene-1-carboxylic acid	1531	-	-	-	-	-	-	22.83	15.05	23.64	-	-	-	-	-	-
21	4-(1-Hydroxy-2-propanyl)cyclohexa-1,3-diene-1-carboxylic acid	1611	-	-	-	-	-	-	29.27	18.35	22.37	-	-	-	-	-	-
22	4-Hydroxy-3,5,6-trimethyl-4-(3-oxobut-1-enyl)cyclohex-2-en-1-one	1790	1.54	-	-	-	-	-	-	-	-	-	-	-	-	-	-
	***Aliphatic hydrocarbons***																
23	Heneicosane	2100	0.73	-	-	0.92	-	0.31	0.86	0.28	0.18	0.94	0.13	-	0.01	0.02	0.19
24	(*Z*)-Tricos-9-ene	2265	6.74	0.53	0.01	2.88	0.01	0.40	2.31	0.03	0.37	5.11	1.25	3.07	-	-	-
25	Tricosane	2300	25.35	5.20	0.89	14.23	3.87	3.82	11.33	3.55	2.16	23.93	4.52	1.34	-	-	-
26	Tetracosane	2400	2.72	0.20	0.10	0.98	3.98	0.10	0.31	0.10	0.02	3.59	0.20	0.67	1.00	1.23	0.91
	***Aliphatic alcohols***																
27	Hexadecan-1-ol	1882	0.08	-	-	-	-	0.39	-	-	0.15	-	0.02	1.31	3.40	0.28	3.33
28	(*Z*)-Octadec-9-en-1-ol	2060	2.18	-	3.67	0.49	1.89	2.41	0.62	0.36	0.61	1.62	0.64	3.81	30.46	18.90	30.42
29	Octadecan-1-ol	2084	1.23	-	2.07	0.02	2.99	1.77	0.20	0.35	0.43	4.82	0.35	4.54	13.97	12.74	13.09
	***Aliphatic aldehydes***																
30	3-Methylpentanal	1028	0.56	0.01	0.04	-	-	-	-	-	0.06	-	0.05	0.08	-	-	-
	***Aliphatic acids***																
31	Octanoic acid	1185	-	0.16	1.64	-	-	1.62	-	0.02	0.27	-	0.14	1.14	-	-	-
32	Decanoic acid	1380	-	-	0.90	-	-	0.62	-	0.01	0.17	-	0.02	0.63	-	-	-
33	Dodecanoic acid	1573	-	-	-	-	-	-	-	-	0.09	-	-	-	-	-	-
34	Hexadecanoic acid	1963	0.58	1.04	3.48	0.74	1.28	4.92	-	0.56	1.37	-	0.88	3.87	0.02	4.92	1.82
35	Oleic acid	2141	2.23	-	-	5.81	-	-	-	-	-	2.87	-	-	-	-	-
36	Octadecanoic acid	2181	19.07	6.68	1.47	17.84	8.47	0.30	10.53	0.58	1.85	32.37	5.12	0.10	0.01	0.22	4.15
	***Aliphatic acid esters***																
37	Ethyl decanoate	1396	-	0.62	0.29	-	0.18	0.39	-	0.24	0.19	-	0.61	0.15	-	-	-
38	Ethyl dodecanoate	1595	-	1.42	-	-	0.49	1.17	-	0.56	0.36	-	1.06	0.05	-	-	-
39	Ethyl tetradecanoate	1794	-	0.75	0.89	-	0.51	0.65	-	0.73	0.37	-	0.54	-	-	-	-
40	Ethyl hexadec-9-enoate	1972	-	0.77	1.25		0.78	1.53	-	0.20	0.23	-	0.40	0.83	-	-	-
41	Ethyl hexadecanoate	1993	0.22	24.98	14.47	0.70	14.88	22.65	-	18.90	12.05	-	21.63	4.24	-	-	-
42	Ethyl linoleate	2157	-	-	-	1.05	-	-	-	-	-	-	-	-	-	-	-
43	Ethyl oleate	2164	7.71	7.33	2.77	28.22	11.72	9.03	4.00	2.31	1.99	9.10	4.95	1.67	-	-	-
44	Ethyl octadecanoate	2194	-	37.17	14.46	3.56	34.34	16.67	-	22.34	9.23	0.81	37.01	11.02	-	-	-
45	Tributyl acetylcitrate	2260	-	-	-	-	-	-	-	-	-	-	-	-	18.08	1.18	14.50
	***Nitrogen compounds***																
46	1-Isoquinolinecarbonitrile *	1446	1.37	0.49	0.93	-	-	-	-	-	-	-	-	-	-	-	-
47	Tryptophol	1769	-	0.92	1.75	-	0.98	1.91	-	0.10	1.04	-	1.68	4.81	-	2.79	1.38
48	1*H*-Indole-3-acetonitrile	1816	3.58	0.02	1.38	-	-	-	-	-	-	-	-	-	-	-	-
49	1*H*-Indole-3-carboxaldehyde	1824	3.30	0.78	5.00	-	-	-	-	-	-	-	-	-	-	-	-
	***Furan derivatives***																
50	Dihydro-5-methyl-2(3*H*)-furanone (γ-Valerolactone)	964	0.14	0.04	0.29	-	-	-	-	-	-	-	-	-	-	-	-
51	2,4-Dihydroxy-2,5-dimethyl-3(2*H*)-furan-3-one	982	0.38	0.10	0.38	0.08	0.04	0.46	0.97	0.16	0.42	0.44	0.10	-	-	-	-
52	Succinic anhydride	1024	-	-	-	-	-	-	-	-	0.22	-	0.20	-	-	-	-
53	2,3-Dihydro-3,5-dihydroxy-6-methyl-4*H*-pyran-4-one	1151	-	-	-	-	-	-	0.10	0.10	0.06	-	-	-	-	-	-

A—honey, B—not boiled mead, C—boiled mead; ^1^ RI: retention indices determined relative to *n*-alkanes (C_9_-C_25_) on the HP-5MS column; *—tentatively identified; names of compound groups were marked in bold/italic.

## Data Availability

Not applicable.

## Sample Availability

The samples are not available from the authors.
